# The Family Physician

**Published:** 1857-08

**Authors:** 


☞ Here is something refreshing to see, a generous tribute to the services, and acknowledgement to the debt of gratitude owed to the family physician, so different from the experiences of flagrant ingratitude which are his daily portion, that we are tempted to copy it. It is taken from the *Advocate and Family Guardian*, published by the Executive Committee of the American Female Guardian Society, and edited by Mrs. Sarah R. I. Bennett.

*The Family Physician.*

Beautiful and benign is that Providential arrangement that "setteth man in families like a flock"—that dots the face of earth with those homes where cluster loving hearts and kindred faces, and from whence flow out the purest and holiest of human sympathies.

How is the whole frame-work of society dependent upon the influences and contributions of the family! The little rills that bubble up by ten thousand hearthstones, and from thence glide noiselessly abroad, are rills of influence whose power for good or evil no human arithmetic can compute. Individually, they may permeate the body social unrecognized. Collectively, they constitute those broad and sweeping rivers whose currents are irresistable, and which bear upon their bosom the barque of Church and State, freighted with all the most precious things of life.

Truly and nobly do they serve their generation who do aught to *educate* those sympathies and influences whose birthplace is Home, or who labor to direct them into those channels where they will most beautify and bless. They serve it *best* who do the *most* in this direction. Parents and teachers are not the only educators of the household. It throws out its tendrils and shoots forth its fibres in every direction, laying hold upon and appropriating for its nourishment and culture all the resources of society.

The Pastor, who appreciates the dignity and importance of the family institution, and who is thoughtful and earnest for the generous and perfected culture there, is a most potent educator. The Family Physician also *may be*, and frequently *is*, one of the educators of the household. What opportunities has he daily and hourly for scattering precious seed! What resources for acquiring and wielding an influence over mind are at his command! He who ministers to the domestic circle in the visitations of disease and bereavement, should be a personage of no inferior position and importance there. A Pastor's influence is often secondary to his. He is not only the dispenser of curative agents, but often *needs to be* of those moral influences which soothe when anguish rends the soul, or sustain when it is ready to sink in hopelessness and dismay. He is the one upon whom the timid lean in their weakness and fears—to whom the dying look and cling as the ebbing tide bears them from the shores of time; for whose footsteps the suffering and anxious watch, and whose changing countenance or expressive demeanor are studied as if he were the oracle of fate.

Unquestioned, he has the *entree* to the inner sanctuary of the home, whatever aspect it wear. To him is the chamber of sickness and seclusion accessible when the footfall of the nearest friend is scarce permitted within its precincts. When a new life, with its wondrous mysteries, unfolds itself within the household circle, and they rejoice over it, and when death with *its* solemn mystery is a pale guest there, and they mourn in bitterness, *he* is *also* there. In the unrestrained raving of delirium—in the prostration and mental weakness disease brings in its train of evils—divested of the guises we wear in health and before the world, do our characters lie open to the inspection of our Physician, and we are dependent upon his offices and his cares.

How important, then, that he be carefully and wisely selected. How many qualities beside professional skill are requisite to constitute such a Family Physician as every household needs! If any profession demands of its members every quality that ennobles and adorns *the man*, surely it is the medical profession.

Many families neither know how to select with discrimination their Physician, nor wisely to avail themselves of all that might profit them in the relationship. Delicacy and propriety, as well as many advantages, attend a *permanency* in the relations between a family and its medical attendant. There is a loss of much that is valuable in a frequent and promiscuous change. He who watches over the health of a family group from the first buddings of infancy up to its maturity, *must* feel an interest and solicitude for that group, which *he could not* who is called in the emergency of to-day and dismissed, when it passes away, to-morrow. A familiarity with its idiosyncracy, and the yearly augmenting professional experience, will conduce to increasing safety and confidence in his practice in the sick room, while he who is for years identified with the family circle will give it that earnest thought, patient care, watchfulness, and sympathy, both in threatened and actual danger, which gold alone could not buy.

A thorough professional education is with most intelligent families an indispensable requisite in their medical adviser. If we are to have charlatans and ignoramuses in any profession, let it not be in this, to whose skill and care we commit, when it is disordered and suffering, this wonderful frame of ours—this delicate mechanism to which is so mysteriously linked the immortal spirit.

Next in importance to professional skill in a Family Physician is, methinks, integrity of principle and genuine piety. Who so frequently as he has need to commit the dying and the bereaved to the tender mercies of the Great Physician, as he rings the knell of their earthly joys in the fearful, reluctant utterance—" No hope—he *must* die !"

A Physician should also be one competent to judge of the moral and mental conditions of his patients, and " to minister," if need be, " to the mind diseased." The *moral prescriptions* he makes—his firmness of nerve, his power of self possession, his talents for social cheer, and the hope and courage he inspires—are frequently of more importance to a sufferer than any draft he may make upon the *Materia Medica.*

It may be that we should more frequently find the Family Physician cultivated in all those qualities that would make his intercourse with the home circle imposing, and as ready to impart information and an *educating influence* as he is to dispense his medicines, if we duly appreciated such qualities, and better understood the mutual obligations of the relation. There is sometimes a backwardness and grudgingness in meeting a Physician's pecuniary claims, which is embarrassing to him and discreditable to those for whom he labors. When sickness lays its hand upon us, and we writhe or faint under it—or when death stands waiting for its prey—we feel that if our Physician will spare no effort to save from the threatened danger, he may " ask of us what he will, even to the half of our kingdom," and it shall cheerfully be given him. But when the portentous cloud has passed from our sky, and our fears have vanished, we sometimes forget his watchings, anxieties, and services, and spend upon our own convenience or extravagancies what is rightfully his.

Physicians as a class are far from a mercenary one. If there was with them no *necessity* in "the bread and butter of life," how many would gladly make their profession subservient only to the relief of suffering humanity and the advancement of science.

To those who most adorn the profession there is accorded a nobler wealth, a purer and finer compensation than gold can proffer. Few comparatively become wealthy as early in life as those in other branches of business requiring equal education, talents and application. The charities of this profession in time, attention, and medicines to the sick poor far exceed those of any other class in the community.

Like other men, the Physician has his susceptibilities to sympathy, and needs sometimes encouragement and appreciation. He needs cooperation with his services, forbearance with his mistakes, and the same charity for his foibles and faults that we feel we have a *right* to expect from him towards our own. Sometimes a Physican is dismissed for some slight mistake, some oversight or omission, which, from the very painfulness of his experience in consequence, he would be on double guard against ever after, and his place is supplied by one who, perhaps, falls into the same or more serious misjudgments, and in his turn is likewise dismissed. None are infallible; therefore should the ill-timed and unnecessary criticism be suppressed with the same consideration we look for him towards the weakness and faults his position enables him to discern in our own domestic circles. Nor should we expect in him creative or omnipotent power; for when the fatal arrow is sped from the quiver of the Almighty, no human hand may stay it.

Like that of other mortals, the Physician's ear must sometimes weary of querulous tones, impatient complaint, and the continual minor key of the invalid's moan. The inmates of that home who, during a morning call from their Family Physician, impart to him of those precious but intangible social influences which elevate, strengthen and cheer, many unwittingly transmit rays of sunshine and hopefulness through the whole round of his day's ministrations. Animal spirits will flag sometimes under constant drafts upon sympathy and patience, and the presence of anxiety and responsibility. Then do such influences do him " good like a medicine."

Irregular meals, loss of sleep, the driving blast or cheerless rain, and the chilly night air, are as repulsive to him as to other men. The mental quiet that takes possession of the business man's mind when he feels that his *day's work is done* the physician can also appreciate, and it would be equally agreeable to him to feel that there was no liability to an interruption of the social chat; no call from the warm, attractive fireside; no necessity for relinquishing slippers and easy-chair, and the enjoyment of a new publication or converse with family or friends.

But if suffering humanity calls, the call is imperative. Personal comfort or social courtesy must be foregone at a moment's notice, and domestic attractions exchanged for the anxious and often repulsive services of the sick-room. Let that family, then, who enjoy the friendship and services of a physician whose qualification meets their moral and physical needs; who is to them an acquisition, *a household blessing*, duly appreciate, love, honor, and sustain him. Let them remember him at the domestic altar, and in many a token and attention of social life, as they do their Pastor; and so regulate the intercourse of the relationship that there may be mutual advantages—reciprocal aids in learning how to live and in preparations for health.—*Peninsular Journal.*

